# The effect of sodium-glucose cotransporter-2 inhibitors on haemoglobin and haematocrit in diabetic kidney disease

**DOI:** 10.1590/1806-9282.20251460

**Published:** 2026-06-26

**Authors:** Süleyman Karaköse, Beyza Doğan, Fatih Ergül, İbrahim Güney

**Affiliations:** 1 University of Health Science, Konya City Hospital, Department of Nephrology – Konya, Turkey.

**Keywords:** Anaemia, Dapagliflozin, Empagliflozin, Chronic kidney disease

## Abstract

**OBJECTIVE:**

The aim of this study was to evaluate the impact of sodium-glucose cotransporter-2 inhibitors on haematological parameters and other clinical markers in this patient population.

**METHODS:**

We retrospectively analyzed the medical records of 150 diabetic patients with chronic kidney disease who initiated sodium-glucose cotransporter-2 inhibitor therapy, comparing their baseline and 3-month post-treatment laboratory and clinical data.

**RESULTS:**

Following 3 months of treatment with sodium-glucose cotransporter-2 inhibitors, mean haemoglobin and haematocrit levels significantly increased, even in patients with an estimated glomerular filtration rate below 60 mL/min/1.73 m^2^. This therapy also led to significant reductions in the spot urine albumin-to-creatinine ratio, as well as in both systolic and diastolic blood pressure.

**CONCLUSION:**

Sodium-glucose cotransporter-2 inhibitor therapy was associated with significant increases in haemoglobin and haematocrit levels in diabetic patients with chronic kidney disease. These findings suggest that sodium-glucose cotransporter-2 inhibitors may represent a potential area requiring further investigation for the treatment of anaemia associated with diabetic chronic kidney disease.

## INTRODUCTION

Diabetic kidney disease (DKD) is the leading cause of chronic kidney disease (CKD) globally. The presence of CKD in diabetic patients is a significant independent risk factor for both cardiovascular disease (CVD) and increased mortality^1^.

Current data show that anaemia is associated with an increased risk of CKD progression, CVD, and mortality. While multiple factors contribute to anaemia as the estimated glomerular filtration rate (eGFR) declines, the primary cause is the impaired secretion of erythropoietin (EPO) due to the loss of functional nephrons^2^.

Sodium-glucose cotransporter-2 (SGLT2) inhibitors regulate blood glucose by promoting renal glucose excretion. Beyond their glucose-lowering effects, they also demonstrate pleiotropic actions that prevent the progression of renal disease and heart failure. Consequently, SGLT2 inhibitors have transitioned from being merely hypoglycaemic agents to being recognized as essential cardiorenal risk-reducing agents in patients with DKD^3^.

While the increase in haemoglobin (Hb) and haematocrit (Hct) from SGLT2 inhibitor therapy is partly attributed to haemoconcentration, this mechanism alone seems insufficient. It is hypothesized that additional factors, such as a positive effect on haematopoiesis, also contribute to this rise^4,5^. Beyond their diuretic effects, SGLT2 inhibitors may influence renal hypoxia and haematopoiesis by increasing Hct levels^5,6^.

A study involving 4,304 participants demonstrated that dapagliflozin, an SGLT2 inhibitor, was associated with an increase in Hct, the correction of anaemia, and a reduced risk of incident anaemia in patients with CKD, irrespective of the presence of type 2 diabetes mellitus (T2DM)^7^.

A study evaluating the effect of GLP-1 receptor agonists and SGLT2 inhibitors on anaemia, involving 13,799 participants, demonstrated that SGLT2 inhibitors reduced the incidence of anaemia^8^.

Data on their impact on Hb and Hct are limited, particularly in patients with DKD. Therefore, this retrospective study was conducted to specifically evaluate the effect of SGLT2 inhibitors on Hb and Hct levels in this patient population.

## METHODS

### Study design and population

This was a single-centre retrospective cohort study conducted at the nephrology clinic. The study protocol was approved by the ethics committee (approval no: 2024/029) and was conducted following the Declaration of Helsinki.

The study population included patients followed at our nephrology outpatient clinic between October 2023 and March 2024. We retrospectively reviewed the files of patients with Stage 1–3 CKD who had initiated treatment with either dapagliflozin 10 mg or empagliflozin 10 or 25 mg. The primary mechanism of SGLT2 inhibition, involving the reduction of glomerular hyperfiltration, is largely established at both dose levels. Furthermore, our initial analysis revealed that when separated, the cohort taking empagliflozin 10 mg and the cohort taking empagliflozin 25 mg had insufficient statistical power to detect meaningful differences in the primary and secondary endpoints (glycated haemoglobin [%HbA1c], eGFR, and urine albumin-to-creatinine ratio [UACR]). Combining the groups enhanced the n (total sample size for the empagliflozin arm) and mitigated the risk of Type II errors (false negatives). The CKD stages were determined based on KDIGO guidelines.

Baseline and 3-month clinical and biochemical data were collected from patient files. The recorded laboratory results included Hb, Hct, mean corpuscular volume (MCV), potassium (K), creatinine, albumin, C-reactive protein (CRP), uric acid, glucose, %HbA1c, and spot UACR. Blood pressure (BP) measurements were recorded following standard guidelines.

From an initial pool of 244 patients, a final cohort of 150 patients was included after applying strict exclusion criteria. Patients were excluded if they had a history of malignancy, gastrointestinal bleeding, kidney transplantation, autoimmune disease, valvular disease, heart failure, active infection, or a deficiency in iron, folic acid, or vitamin B12.

In our retrospective cohort of patients with CKD stages 1, 2, and 3, the dataset did not include cases of iron deficiency anaemia, iron supplementation, or ESA treatment at baseline or during follow-up.

Biochemical and clinical data were collected from each patient’s files at two time points: baseline and 3 months after treatment. The recorded laboratory results included Hb, Hct, and MCV; potassium (K), creatinine, albumin, and uric acid; CRP; glucose and %HbA1c; and spot UACR. Additionally, BP measurements were routinely recorded at each patient visit, following standard clinical guidelines^9^.

### Statistical analysis

Statistical analyses were performed using SPSS version 22.0 (Statistical Package for Social Sciences). The Kolmogorov-Smirnov test was used to assess for normality. Data are presented as mean±standard deviation for normally distributed variables and as median (minimum–maximum) for skewed variables. For pairwise comparisons, the paired-samples t-test was used for normally distributed data, while the Wilcoxon signed-rank test was performed for non-normally distributed data. p<0.05 was considered statistically significant.

## RESULTS

A total of 150 patients were included in the study, with 101 patients (67.3%) receiving dapagliflozin and 49 patients (32.7%) receiving empagliflozin. The mean age of all patients was 60.23±10.2 years, with an age range of 27–82 years.

At the beginning of the SGLT2 inhibitor treatment, the mean Hb and Hct levels were statistically significantly lower than at the third month (13.54±1.74 g/dL vs. 14.03±1.77 g/dL, p<0.001; and 41.09±5.04% vs. 42.99±4.63%, p<0.001, respectively). White blood cell (WBC) counts were not statistically different. Fasting glucose levels did not show a statistically significant difference, but HbA1c values were statistically lower at the third month (7.6 vs. 7.5, p<0.001). The spot UACR was also significantly lower at the third month (204.5 vs. 81, p<0.001). Additionally, there was a decrease in systolic blood pressure (SBP) and diastolic blood pressure (DBP) levels with statistically significant differences at the third month (147 mmHg vs. 135 mmHg, p<0.001; and 80 mmHg vs. 76 mmHg, p<0.001). As expected, the creatinine value was lower (1.1 mg/dL [0.49–2.14] vs. 1.20 mg/dL [0.55–2.62]) and eGFR value was higher (60.5 [30–130] mL/dk/1.73 m^2^ vs. 58.5 [21–133] mL/dk/1.73 m^2^) at baseline when compared to the third month’s values with statistically significant differences (p<0.001 and p<0.001, respectively) (Table 1).

**Table 1:** The laboratory and clinical results of all patients.

Parameters	Baseline (n=150)	At the third month (n=150)	p
Cre (mg/dL)	1.1 (0.49–2.14)	1.20 (0.55–2.62)	*<0.001*
eGFR (mL/min)	60.5 (30–130)	58.5 (21–133)	*<0.001*
K+ (mmol/l)	4.8 (3.2–6.0)	4.6 (3.5–5.8)	0.06
Hb (g/dL)	13.54±1.74	14.03±1.77	*<0.001*
Hct	41.09±5.04	42.99±4.63	*<0.001*
MCV	86.66±4.71	85.95±5.54	0.028
WBC	8,325 (864–16,200)	8,065 (1,040–16,640)	0.656
Glucose (mg/dL)	143 (59–350)	141 (61–418)	0.358
HbA1c	7.6 (5–14.9)	7.5 (5–12.6)	*<0.001*
CRP	2.0 (0–31)	2.0 (0–99)	0.124
Albuminuria (mg/dL)	204.5 (0–5,480)	81 (0–6,259)	*<0.001*
SBP (mmHg)	147 (99–194)	135 (100–184)	*<0.001*
DBP (mmHg)	80 (60–121)	76 (50–109)	*<0.001*

Cre: creatinine; GFR: estimated glomerular filtration rate; K^+^: potassium; MCV: mean corpuscular volume; Hb: haemoglobin; Hct: haematocrit; WBC: white blood cell; CRP: C-reactive protein; SBP: systolic blood pressure; DBP: diastolic blood pressure. Italic values indicate statistical significance (p<0.05).

Hb and Hct levels increased significantly in both the dapagliflozin and empagliflozin groups. HbA1c levels were lower at the third month for patients on dapagliflozin 10 mg (7.92±1.85 vs. 7.61±1.48, p=0.007) and empagliflozin 25 mg (7.7 vs. 7.7, p=0.010). The SBP levels were statistically lower at the third month in all patient groups (Table 2).

**Table 2:** The laboratory and clinical results of patients according to the drugs used.

	Dapagliflozin (n=101)	Empagliflozin 10/25 (n=49)	Empagliflozin 10 (n=23)	Empagliflozin 25 (n=26)
Cre				
Baseline	1.10 (0.56–2.14)	1.15±0.32	1.23±0.33	1.09±0.33
Third month	1.22 (0.55–2.62)	1.21±0.38	1.24±0.34	1.19±0.40
p	*<0.001*	*0.019*	0.748	*0.002*
eGFR				
Baseline	61 (30–118)	57 (30–130)	54 (30–130)	57.5 (34–109)
Third month	58 (21–113)	59 (26–133)	52 (30–133)	59.5 (29–104)
p	*<0.001*	0.114	0.917	0.290
K+				
Baseline	4.75±0.48	4.71±0.47	4.50 (4.0–5.5)	4.95 (3.2–5.4)
Third month	4.61±0.49	4.66±0.45	4.4 (3.7–5.3)	4.90 (4.2–5.5)
p	*0.001*	0.390	0.300	0.406
Hb				
Baseline	13.51±1.68	13.66±1.84	13.31±1.92	13.96±1.76
Third month	13.96±1.71	14.20±1.87	13.87±1.98	14.50±1.75
p	*<0.001*	*<0.001*	*0.003*	*0.001*
Hct				
Baseline	40.94±5.25	41.41±4.60	40.94±4.79	41.83±4.47
Third month	42.84±4.52	43.30±4.88	42.46±5.24	44.04±4.51
p	*<0.001*	*<0.001*	*0.010*	*<0.001*
HbA1c				
Baseline	7.92±1.85	8.1±1.43	8.3 (5.6–11.3)	8.3 (6.0–10.8)
Third month	7.61±1.48	7.74±1.38	7.5 (5.6–12.6)	7.7 (6.1–9.9)
p	*0.007*	*0.007*	0.217	*0.010*
Albuminuria				
Baseline	259 (0–4,817)	80 (0–5,480)	51.5 (0–3,417)	102 (0–5,480)
Third month	111 (0–6,259)	10 (0–4,490)	3 (0–4,490)	39 (0–1,885)
p	*<0.001*	*0.005*	0.209	*0.005*
SBP				
Baseline	147.1±20.2	147.7±20.8	144.4±22.9	150.6±18.8
Third month	135.5±18.6	137.4±21.3	131.9±23.7	142.3±18.0
p	*<0.001*	*<0.001*	*<0.001*	*0.019*
DBP				
Baseline	81.4±10.6	80.5±8.1	78.7±5.9	82.1±9.4
Third month	76.7±10.3	76.3±9.1	73.8±7.5	78.5±0.9
p	*<0.001*	*0.003*	*0.003*	0.120

Cre: creatinine; GFR: estimated glomerular filtration rate; K^+^: potassium; Hb: haemoglobin; Hct: haematocrit; SBP: systolic blood pressure; DBP: diastolic blood pressure. Italic values indicate statistical significance (p<0.05).

In the subgroup of patients with baseline eGFR between 25 and 60 mL/min/1.73 m^2^, Hb and Hct values increased significantly in both dapagliflozin (13.15±1.57 vs. 13.49±1.72, p=0.010; and 40.31±4.53 vs. 41.60±4.48, p=0.001) and empagliflozin groups (13.12±1.55 vs. 13.73±1.67, p<0.001; and 39.77±3.99 vs. 41.94±4.50, p<0.001). SBP levels were also significantly lower at the third month in both subgroups (150.6±21.3 vs. 137.9±18.8, p<0.001 for dapagliflozin; and 149.6±20.9 vs. 138.2±20.9, p=0.002 for empagliflozin) (Table 3).

**Table 3:** The laboratory results of the patients who had 25<eGFR<60 mL/dk/1.73 m^2^.

Parameters	Baseline	At the third month	p
Cre			
Dapagliflozin (n=45)	1.42±0.29	1.53±0.41	*0.004*
Empagliflozin 10/25 mg (n=27)	1.40 (1.0–1.78)	1.54 (0.9–1.91)	*0.046*
eGFR			
Dapagliflozin (n=45)	47 (30–58)	41 (21–83)	*0.008*
Empagliflozin 10/25 mg (n=27)	50 (30–58)	43 (29–82)	0.118
Potassium			
Dapagliflozin (n=45)	4.88±0.53	4.66±0.58	*0.003*
Empagliflozin 10/25 mg (n=27)	4.7 (3.2–5.4)	4.66 (3.7–5.5)	0.198
Hb			
Dapagliflozin (n=45)	13.15±1.57	13.49±1.72	*0.010*
Empagliflozin 10/25 mg (n=27)	13.12±1.55	13.73±1.67	*<0.001*
Hct			
Dapagliflozin (n=45)	40.31±4.53	41.60±4.48	*0.001*
Empagliflozin 10/25 mg (n=27)	39.77±3.99	41.94±4.50	*<0.001*
Albuminuria			
Dapagliflozin (n=45)	318.5 (0–4,817)	120.5 (0–6,259)	*0.003*
Empagliflozin 10/25 mg (n=27)	99.5 (0–3,417)	27.5 (0–4,490)	0.390
SBP			
Dapagliflozin (n=45)	150.6±21.3	137.9±18.8	*<0.001*
Empagliflozin 10/25 mg (n=27)	149.6±20.9	138.2±20.9	*0.002*
DBP			
Dapagliflozin (n=45)	80.9±12.2	75.3±10.9	*0.001*
Empagliflozin 10/25 mg (n=27)	78.4±8.0	76.7±9.2	0.333

Cre: creatinine; GFR: estimated glomerular filtration rate; Hb: haemoglobin; Hct: haematocrit; SBP: systolic blood pressure; DBP: diastolic blood pressure. Italic values indicate statistical significance (p<0.05).

## DISCUSSION

Our study found that SGLT2 inhibitor treatment is associated with a statistically significant increase in both Hb and Hct levels in patients with DKD. This effect was also observed in the subgroup of patients with an eGFR below 60 mL/min/1.73 m^2^. The small but statistically significant increase observed in our study can be interpreted as suggestive evidence that the treatment is associated with activation of the haematopoietic pathway. This finding contributes to the existing body of literature by indicating a potential link between SGLT2 inhibitor use and modest haematological changes, which may be relevant to the discussion of managing anaemia, particularly in the context of progressive CKD. Due to the retrospective nature of our study, we were unable to measure routine follow-up EPO levels; consequently, we cannot definitively establish the contribution of SGLT2 inhibitors to EPO elevation, thus precluding strong causal inferences. However, despite this limitation, we believe our results are suggestive of a positive influence of SGLT2 inhibitors on anaemia-related parameters.

Furthermore, we observed significant reductions in the spot UACR, HbA1c levels, and SBP. Also, our study reported an initial increase in serum creatinine and subsequent decrease in eGFR, which reflect the expected and desired pharmacological effect of SGLT2 inhibitors and do not indicate a genuine worsening of renal function. SGLT2 inhibitors exert their effect by modulating the tone of the afferent arteriole and reducing glomerular hyperfiltration. This action leads to a transient and mild initial decline in eGFR (10–15%) due to changes in renal haemodynamics. These findings are consistent with the results of landmark trials such as DAPA-CKD, CREDENCE, and EMPA-KIDNEY, which have established the cardiorenal protective benefits of SGLT2 inhibitors^8,9,10^. Ultimately, our results underscore the importance of SGLT2 inhibitors in the comprehensive management of DKD, as controlling key risk factors such as hyperglycaemia, hypertension, proteinuria, and anaemia is critical for slowing disease progression^9,11,12^. According to current literature, the beneficial effects of SGLT2 inhibitors on DKD are primarily attributed to their actions on proteinuria, hypertension, and hyperglycaemia^13,14^. Although anaemia is a recognized factor in the progression of DKD, the effect of SGLT2 inhibitors on Hb and Hct levels in this specific patient population has not been fully elucidated. In light of this, our study aimed to specifically investigate the impact of SGLT2 inhibitors on these haematological parameters in patients with chronic DKD.

In different well-designed studies on patients with heart failure, an increase in Hb and Hct levels has been reported after SGLT2 inhibitor treatment^15,16,17,18^. At the first glance, this rise in Hct might be attributed solely to haemoconcentration caused by the diuretic effects of SGLT2 inhibitors. However, findings from the DAPA-HF study challenge this simple explanation. The study reported a consistent and similar degree of increase in Hct levels in patients both with and without diabetes mellitus. Furthermore, the rise in Hct seemed to peak after approximately 4 months of treatment, which is a considerably longer duration than what would be expected from the diuretic effect alone. This suggests that additional mechanisms, beyond haemoconcentration, are likely contributing to these haematological changes^19^. Also, clinical studies have demonstrated that serum EPO levels increase within 4 weeks of initiating SGLT2 inhibitor therapy^20,21^. It is therefore hypothesized that SGLT2 inhibitors promote EPO secretion, which subsequently increases Hct and contributes to improved outcomes in heart failure^19,21^. Furthermore, it has been hypothesized that SGLT2 inhibitor-mediated activation of hypoxia-inducible factor 2 (HIF-2) may enhance the secretion of EPO from renal interstitial cells. This mechanism is considered a potential cause for the observed increase in Hb and Hct levels^22,23,24^.

There is limited data in the literature on the effect of SGLT2 inhibitors on EPO levels in CKD patients. Our retrospective study was limited by the inability to collect these data. This is a notable limitation, as the decrease in EPO levels due to nephron loss is a significant cause of anaemia in CKD, particularly when the eGFR falls below 60 mL/min/1.73 m^2^. Despite this, an increase in both Hb and Hct was observed in our study cohort, even among patients with an eGFR below this threshold. In a recent post hoc mediation analysis of the EMPA-REG OUTCOME trial, Wanner et al. reported that an increase in both Hb and Hct was the strongest mediator of empagliflozin’s kidney benefits in patients with T2DM and CVD^25^. The EMPA-REG OUTCOME trial demonstrated that both the 10 mg and 25 mg doses of empagliflozin provided consistent risk reductions for major cardiovascular events and the progression of nephropathy. This finding suggests that the core protective mechanisms of SGLT2 inhibitors—specifically, the reduction of glomerular hyperfiltration and the mitigation of renal cortical hypoxia—are largely saturated at both dose levels. Furthermore, the existing literature indicates that the benefits of SGLT2 inhibitors in correcting anaemia can often be achieved even with lower doses, without significantly enhanced benefit from higher dosages. Consequently, this established pharmacological equivalence strongly supports our decision to combine the 10 mg and 25 mg empagliflozin cohorts into a single therapeutic category for the analysis of renal and haematological endpoints in our study.

Major trials did not report Hb and Hct data for patients with an eGFR below 60 mL/min/1.73 m^2^. Our study aimed to address this gap and observed an increase in the relevant levels even within this patient group below the eGFR threshold. This finding, coupled with Milton Packer’s research on SGLT2 inhibitors activating HIF-2α, suggests that these drugs could become a future treatment option for anaemia associated with chronic DKD^26^. Further studies are needed to validate the effect of SGLT2 inhibitors on anaemia in patients with CKD, particularly in those with an eGFR below 60 mL/min/1.73 m^2^. This specific subgroup warrants further investigation to confirm these findings.

The primary limitations of our investigation stem from its retrospective, single-centre design and the characteristics of the study cohort. Specifically, the absence of a dedicated control group restricts our ability to definitively attribute the observed small changes in Hb/Hct solely to SGLT2 inhibitor therapy, rather than to routine clinical management or other unmeasured factors (EPO). Crucially, our population was largely non-anaemic at baseline (mean Hb>13 g/dL) and the short duration of follow-up (3 months) limits the generalizability of our findings regarding sustained, clinically meaningful long-term effects.

In conclusion, our results indicate a potentially favourable signal on haematological parameters; however, we agree that this signal must be viewed as an area requiring further investigation rather than an established indication. Consequently, we advocate for the necessity of large-scale, high-sample, prospective, controlled studies utilizing real-world data from truly anaemic CKD populations. Such future research is essential to conclusively determine the causal role, magnitude, and clinical utility of SGLT2 inhibitor use in the management of CKD-related anaemia.

## Data Availability

The datasets generated and/or analyzed during the current study are available from the corresponding author upon reasonable request.
